# Effects of* Angelica* Extract on Schwann Cell Proliferation and Expressions of Related Proteins

**DOI:** 10.1155/2017/6358392

**Published:** 2017-07-18

**Authors:** Xiaowen Jiang, Lin Liu, Binqing Zhang, Ziyin Lu, Lu Qiao, Xinxin Feng, Wenhui Yu

**Affiliations:** ^1^College of Veterinary Medicine, Northeast Agricultural University, Harbin 150030, China; ^2^Key Laboratory of the Provincial Education, Department of Heilongjiang for Common Animal Disease Prevention and Treatment, Northeast Agricultural University, Harbin 150030, China

## Abstract

The present study investigated the effects of* Angelica* extract (AE) on Schwann cell proliferation and expressions of related proteins, including brain derived neurotrophic factor (BDNF), neural cell adhesion molecule (NCAM), and proliferating cell nuclear antigen (PCNA). Proliferation activity and cell cycles of SCs were evaluated by MTT assay and flow cytometry methods, respectively, after 12 h treatment of AE at different concentrations (62.5, 125, 250, 1000, 2000, 4000, and 8000 mg/L). SCs were treated by 500, 1000, and 2000 mg/L AE for 24 h or 48 h; the related genes mRNA and proteins expressions in SCs were detected by quantitative real-time reverse transcription-polymerase chain reaction (RT-PCR) and enzyme-linked immunosorbent assay (ELISA) kit. At the concentration range of 125–2000 mg/L, the SC proliferation was induced by AE in a dose-dependent manner, especially 1000 and 2000 mg/L; cells in drug-treated groups showed the most increase. Cells counts were ascended significantly in (G2/M + S) phase compared to control group. BDNF, NCAM, and PCNA protein expressions significantly increased at drug-treated groups. Relative genes mRNA expressions levels were also significantly higher compared to control group. The results indicated that AE facilitated SC proliferation and related genes and proteins expressions, which provided a basic guideline for nerve injury repair in clinic.

## 1. Introduction

Schwann cells (SCs) are unique glial cells in the peripheral nervous system and they play vital roles in generation, development, morphology, maintaining function, and other aspects of peripheral nerves [1]. SCs are important for the regeneration and repairment of peripheral nerve injury [2]. When distal site of injured nerve witnessed Waller degeneration, SCs began to proliferate and then participated in swallowing degeneration of axons and myelin debris forming a longitudinal continuous cell cord (B*ǜ*ngner's band), which guided the growth of regenerating axons. SCs are essential for regeneration of microenvironment [3], which is the important condition for neuronal survival and neurite growth. SCs have nutrition effects on neuron due to synthetic neurotrophic factors, and they also contribute to generating neurite growth factors [4]. SCs secrete a variety of neurotrophic factors, such as nerve growth factor (NGF), fibroblast growth factor (FGF), BDNF, NCAM, PCNA, and ciliary neurotrophic factor (CNTF). These factors play major roles in peripheral nerve cell growth, development, regeneration, and maintaining normal nerve cells alive, and they are beneficial to axons regeneration and myelination [5, 6]. Lack of neurotrophic factors may cause neurological diseases and failure of nerve regeneration [7].

*Angelica*, Umbelliferae* Angelica sinensis* (Oliv.) Diels dried roots, is regarded as a traditional natural medicine for invigorating the circulation of blood. Currently, many researches showed that* Angelica* had a variety of pharmacological effects, including anti-inflammatory [8], anticancer [9], wound healing [10], and nerve regeneration [11] effects. Nerve injury is a common clinical disease; as the research on the treatment of the disease gradually increases, more and more natural plants were applied in peripheral nerve repair. It was reported that natural plants showed fewer side effects and minimize drug resistance [12]. Some studies have demonstrated the effects of* Angelica* on nerve repair. It was also reported that AE attenuated neuropathic pain, which is associated with proinflammatory cytokines such as tumor necrosis factor-*α* (TNF-*α*), interleukin-1*β* (IL-1*β*) and interleukin-6 (IL-6), TRPV1, and p-ERK in peripheral nervous pain systems [13]. Some findings suggested that* Angelica* injection improved the sciatic nerve crush injury, and the mechanism might be through the increase of BDNF and NGF protein expressions [14]. Angelica dahuricae radix decreased the levels of TNF-*α*, IL-1*β*, IL-6, inducible nitric oxide synthase (iNOS), and cyclooxygenase-2 (COX-2) in a lipopolysaccharide- (LPS-) activated microglial cell line and provided neuroprotection by alleviating inflammation and oxidative stress [15]. Another research elucidated that AE promoted PC12 cell proliferation in vitro assay [16]. The AE contains volatile oil, organic acids, polysaccharides, flavonoids, and other ingredients [17]. These findings showed that* Angelica* exerted a positive-effect in nerve injury repair. However, the studies of AE in vitro assays are not common, and particularly effects of AE on SCs (important peripheral nerve cells) have not been reported. In the present study, we investigated the effects of AE on SC proliferation and cycle, protein, and mRNA expressions of BDNF, NCAM, and PCNA, which are related to peripheral nerve repair [18–20]. The detection of these neural factors in AE-treated SCs will provide a mechanistic framework for further studies of the use of AE as an effective treatment for peripheral nerve injury.

## 2. Materials and Methods

### 2.1. Equipment and Reagents

HB050 type inverted phase contrast microscope was manufactured by Zeiss company; K330 refrigerated centrifuge was manufactured by Sigma Corporation; iMark550 microplate reader was manufactured by Bio-Rad company; BD flow cytometer was manufactured by American companies. GeneQuant 1300 spectrophotometer was manufactured by General Electric Company. LightCycler 96 was produced by Roche in Switzerland.

AE was purchased from Baoji FangSheng Biological Development Co., Ltd. Rat SCs (RSC96) were obtained from China Cell Line Repository. Dulbecco Modified Eagle Medium (DMEM) was purchased from HyClone company. Fetal Bovine Serum (FBS) was purchased from Sijiqing company in China. MTT and Propidium Iodide (PI) reagents were purchased from American Sigma company. Rat BDNF, NCAM, and PCNA-ELISA kits were produced by Beyotime Institute of Biotechnology in JiangSu. First-strand cDNA synthesis kit was purchased from Thermo Scientific Company. All primers were designed and synthesized by Invitrogen Biotechnology Co., Ltd. (Shanghai, China).

### 2.2. Cell Toxic Assays

The fifth passage of SCs was used for experiments, and the trypsinized cells were diluted with Dulbecco Modified Eagle Medium (DMEM) containing 10% Fetal Bovine Serum (FBS) to cell suspension at a concentration of 5.0 × 10^4^ cells/mL. SCs were seeded in 96-well culture plates and 200 *μ*L cell suspension was added to every well. Cells were incubated at 37°C under 5% CO_2_ for 24 h, then the medium was removed and DMEM without FBS was added per well, and cells were hungered for 12 h and were randomly divided into control group and drug-treated groups. The control group and drug-treated groups were cultured dividedly by DMEM containing 10% FBS without or with the medium containing indicated concentrations (62.5, 125, 250, 1000, 2000, 4000, and 8000 mg/L) of AE for 12 h at 37°C. Cell viability was measured by MTT assay. Five parallel wells were set up for a group and the MTT assay was repeated 3 times.

### 2.3. The Effects of AE on Proliferation of SCs

SCs growth was interfered with by AE with the indicated concentration that increased SC proliferation positively. SCs treated by DMEM containing 10% FBS acted as control, and all groups are set up with 7 parallel wells. The experiments were performed for duplicating at least three times. Cells were cultured for 12 h, 24 h, 36 h, and 48 h. The effects of AE on cell proliferation were evaluated by MTT assay as previously described.

### 2.4. The Effects of AE on the Cell Cycles of SCs

SCs in logarithmic phase were trypsinized and centrifuged; SCs were diluted with DMEM containing 10% FBS and seeded in 25 mm^3^ glass screw-cap cell bottle at a density of 2.0*∗*10^5^ cells/mL. After 24 h of incubation at 37°C, the medium was removed and then cells were treated with DMEM for 12 h. In the drug-treated groups, AE mixed with DMEM containing FBS was added to every well at a concentration that promoted cell proliferation, and the cells in control group were only treated by DMEM containing FBS. The supernatant was discarded after incubating for 24 h and 48 h. The cell cycle of each group was detected by flow cytometry. Modfit LT software was used for analyzing cell cycle of each group. The experiments were performed for repeating at least three times.

### 2.5. Effects of AE on Expressions of BDNF, NCAM, and PCNA in SCs

SCs growths were interfered with by AE with the concentrations which promoted SC proliferation, and then cells were cultured for 24 h and 48 h, respectively. Then the number of SCs was counted, and the culture supernatants were collected to determine the amount of BDNF, NCAM, and PCNA secreted by the cultured SCs. Cell culture supernatants were centrifuged and assayed by using an ELISA kit, following the manufacturer's instructions; cells were trypsinized and cell supernatants were treated according to manufacturer's instruction of ELISA. The protein samples were stored at −80°C until being assayed. The samples were used to measure BDNF, NCAM, and PCNA. The experiment was repeated three times.

### 2.6. Effects of AE on Gene Expression of BDNF, NCAM, and PCNA

SCs were treated by AE for 48 h at concentrations of 500, 1000, and 2000 mg/L, respectively. Total RNA was isolated from SCs using TRIzol reagent according to the manufacturer's instructions. The RNA concentration was determined using a GeneQuant 1300 spectrophotometer, and the purity of RNA was determined using the 260/280 nm absorbance ratio. The *A*260/*A*280 ratio of the RNA samples was 1.8–2.0. First-strand cDNA was synthesized from 5 *μ*g of total RNA according to the manufacturer's instructions. All primers were designed and synthesized by Invitrogen Biotechnology Co., Ltd. The primers used are shown in Table 1.

RT-PCR was performed using a LightCycler 96. The annealing temperatures and the thermocycling conditions for the target genes were as follows: BDNF (57.9°C, 35 cycles), NCAM (57.9°C, 35 cycles), PCNA (57.9°C, 35 cycles), and *β*-actin (59°C, 35 cycles). The standard PCR conditions were as follows: 94°C for 30 s and 45 cycles of 94°C for 5 s, a variable annealing temperature for 15 s and 72°C for 10 s. The experiments were performed in triplicate and repeated at least three times. Mean Ct values were used to calculate the relative expression levels of the target genes for the experimental groups, relative to those in the negative control group. The relative expression of target genes was obtained using the 2^−ΔΔCt^ formula using *β*-actin as a housekeeping gene.

### 2.7. Statistical Analyses

All data were presented as mean values ± standard deviation, and statistical analysis was performed using a SPSS 13.0 statistical package. The multiple comparisons of data were performed by one-way ANOVA followed by Dunnett's test. Significant differences were defined at values of *P* < 0.05 and extreme significant differences were defined at values of *P* < 0.01.

## 3. Results

### 3.1. AE Stimulated SC Proliferation at Indicated Concentration

**Figure 1:**
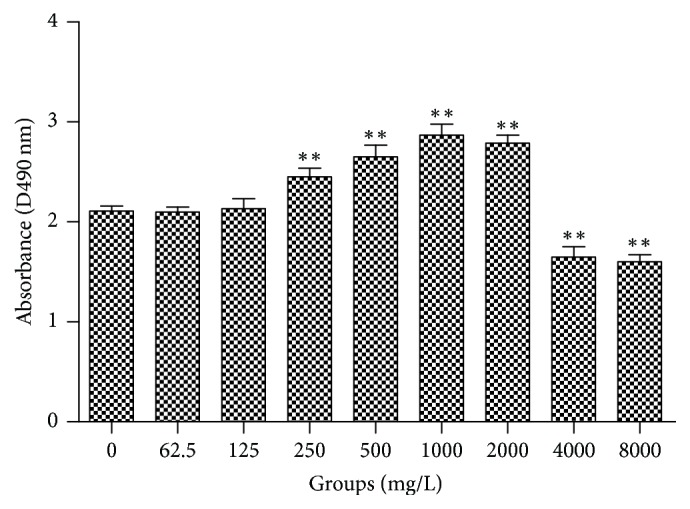
Cell toxic effects of AE on SCs (*n* = 6, ±s). This photograph showed SC viability after 12 h treatment with different concentrations of AE. DMEM with 10% FBS was used as control. Notes: ^*∗*^*P* < 0.05, ^*∗∗*^*P* < 0.01, versus the control group (Student's *t*-test).

SC proliferation is important for the healing of nerve injury. Cell toxic assays were preformed to explore effects of AE on SC proliferation. According to Figure 1, result of cell toxic assay showed that, after treatment with different concentrations of AE, the concentration range of AE which was conducive to the growths of SCs was 125–2000 mg/L and showed dose-dependent manner, and when concentration of AE was lower than 125 mg/L, the SCs growths in drug-treated groups and control group had no significant differences. However, when AE concentration was higher than 4000 mg/L, SC proliferation was inhibited significantly compared to the control group (*P* < 0.05). Therefore, these findings indicated that AE stimulated SC proliferation at appropriate concentration range and the optimal concentration was 1000 mg/L.

### 3.2. AE Promoted SC Proliferation at Different Time Points

**Figure 2:**
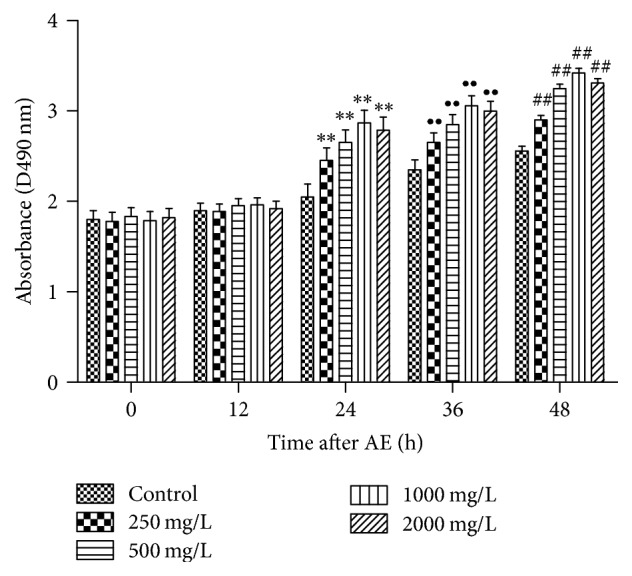
Drug concentration and application time effects on SC proliferation (*n* = 12, ±s). The viability of SC at 0 h, 12 h, 24 h, 36 h, and 48 h was assessed after being treated with different concentrations (250~2000 mg/mL) of AE. Notes: ^*∗∗*, ∙∙, ##^*P* < 0.01 versus control group at the same time (Student's *t*-test).

Through the cytotoxicity test, the effective concentrations of AE were determined. To further study the effects on SC proliferation at different time points with treatment of AE, cell proliferation test was performed. As shown in Figure 2, when AE concentration was in the 250–2000 mg/L range, AE promoted SC proliferation after treating for 24 h, 48 h, and 72 h; at a concentration of 500–2000 mg/L, cell proliferation in different groups was significantly increased by comparison with control group (*P* < 0.05); when the concentrations were 1000 and 2000 mg/L, drug-treated groups showed the extremely significant difference compared to control group (*P* < 0.01).

### 3.3. The Effects of AE on the Cell Cycles of SCs

**Figure 3:**
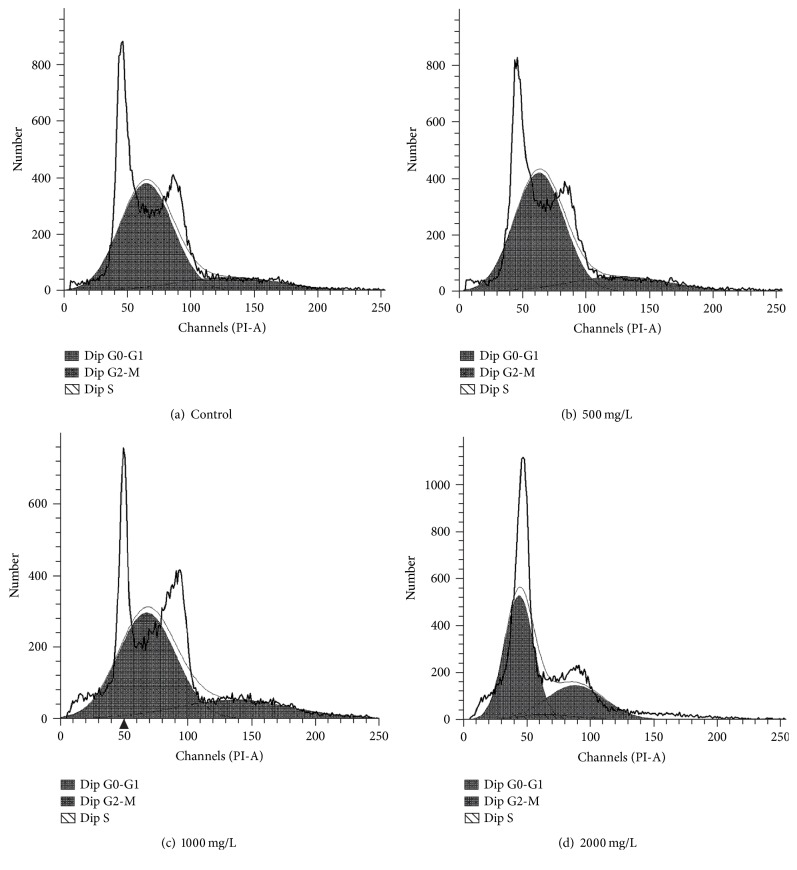
Different concentrations of AE exerted an influence on the SCs cycles (24 h). SCs were gathered at 24 h after being treated with 0, 500, 1000, and 2000 mg/mL AE, and then SCs cycles were detected using flow cytometry in sequence. The results were shown, respectively, in (a), (b), (c), and (d).

**Figure 4:**
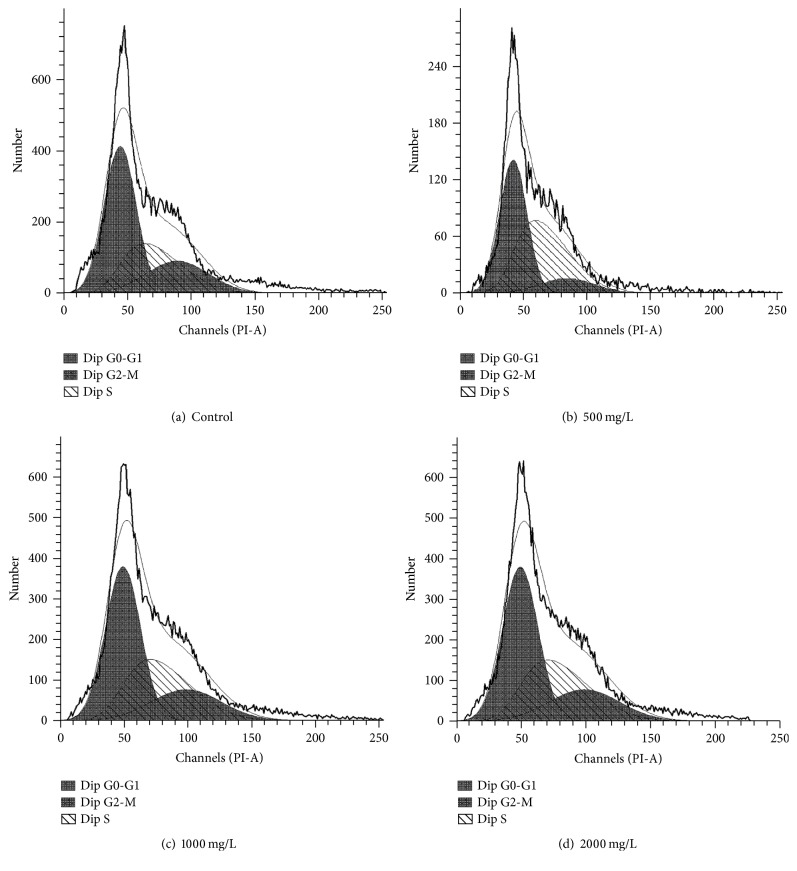
Different concentrations of AE exerted an influence on the SCs cycles (48 h)/SCs were gathered at 48 h after being treated with 0, 500, 1000, and 2000 mg/mL AE, and then SCs cycles were detected using flow cytometry in sequence. The results were shown, respectively, in (a), (b), (c), and (d).

**Figure 5:**
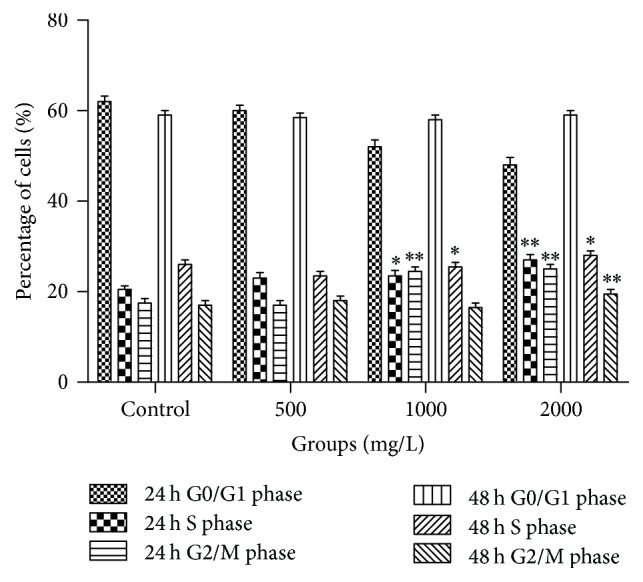
Effects of different concentrations of AE on SCs cycles (*n* = 5, ±s, %). Modfit LT software was used for analyzing cell cycle of each group after 24 or 48 h treatment with 500, 1000, and 2000 mg/mL of AE. Notes: ^*∗*^*P* < 0.05 and ^*∗∗*^*P* < 0.01, as compared with the control group (Student's *t*-test).

We examined the cell cycle in AE-treated SCs. The G2 phase is the late stage of DNA synthesis and the M phase is the stage of mitosis, and they all reflect the state of cell proliferation to some extent. As shown in Figures 3 and 4, SCs were treated by 500, 1000, and 2000 mg/L concentrations of AE for 24 h or 48 h, compared with control group, and the results showed that cells percentage composition had differences in G0/G1, S, and G2/M phase, respectively. Moreover, we found that the proliferation index (PI = S + G2/M) was significantly increased. The percentage of drug-treated cells in (G2/M + S) phase was higher than control group (*P* < 0.01), and this finding showed that AE activated synthesis of DNA, which leads to cell proliferation (Figure 5).

### 3.4. AE Increased BDNF, NCAM, and PCNA Protein Expressions in SCs

**Figure 6:**
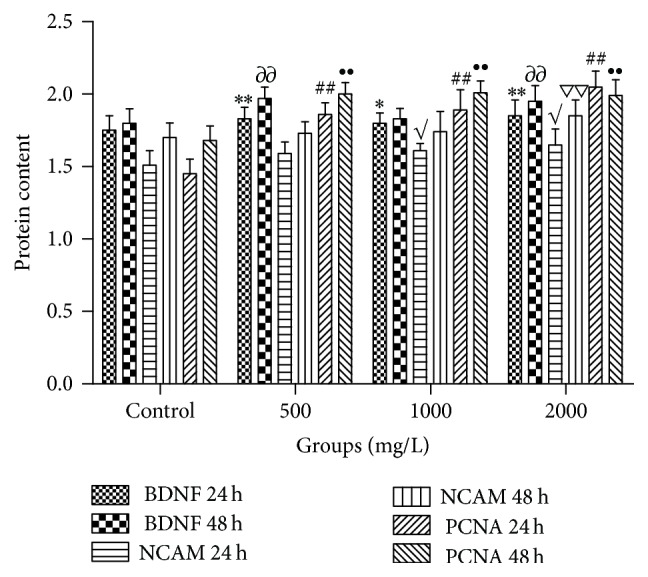
Effects of different concentrations of AE on expressions of the relative secretion cytokine of SCs (*n* = 5, ±s). BDNF, NCAM, and PCNA expressions were detected by ELISA kits at 24 and 48 h after stimulation without or with indicated concentrations of AE. Notes: single symbol such as “*∗*” means *P* < 0.05 compared with control; double symbols such as “*∗∗*” mean *P* < 0.01 compared with control.

Expressions of BDNF, NCAM, and PCNA protein are important for regeneration of nerve. The ELISA assays were performed. When AE concentration was 1000 mg/L, BDNF and PCNA contents in cells were checked after incubating for 24 h, and the results showed significant difference compared with control group (*P* < 0.05); the expression of PCNA in cells cultured for 48 h were detected, and these results revealed that drug-treated cells expressed more PCNA protein compared with cells in control group (*P* < 0.01). 2000 mg/L of AE-treated cells for 24 h and expressions of BDNF and PCNA were extremely significant higher than control group (*P* < 0.01). NCAM was upregulated significantly compared with the control group (*P* < 0.05). After incubation of 48 h, expressions of BDNF, NCAM, and PCNA increased obviously compared with the control group (*P* < 0.01), and all were shown in Figure 6.

### 3.5. AE Promoted Relative Genes Expressions in SCs

**Figure 7:**
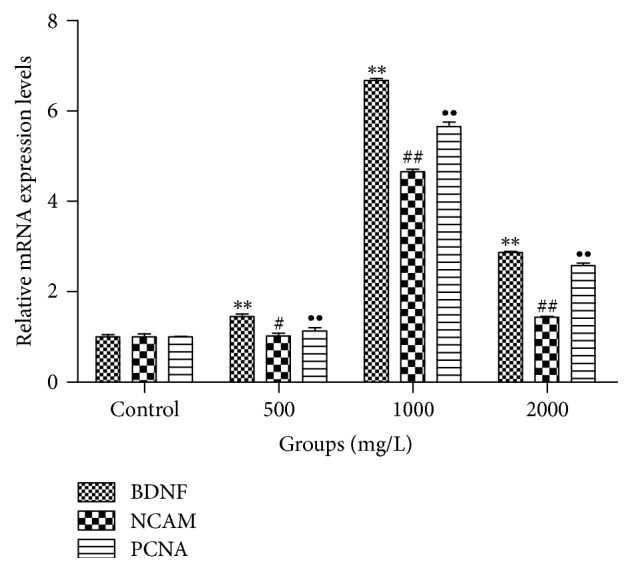
Effects of different concentrations of AE on mRNA expressions of BDNF, NCAM, and PCNA in SCs (*n* = 4, ±s). AE interfered with SCs growths for 48 h at concentrations of 500, 1000, and 2000 mg/L, respectively; gene expressions of BDNF, NCAM, and PCNA were checked by RT-PCR. Results were presented as the folds change compared with untreated cells. Notes: ^*∗*, #, ∙^*P* < 0.05 compared with control group; ^*∗∗*, ##, ∙∙^*P* < 0.01 compared with control group (Student's *t*-test). “*∗*” and “∙” refer to differences in each gene expression between control group and drug-treated groups.

To further examine the effects of AE on BDNF, NCAM, and PCNA, gene expressions were checked. 500, 1000, and 2000 mg/L of AE interfered with cells growths for 48 h, RNA purity was detected, and generally the extracted RNA are available when the detection results belong to 1.8–2.0. As shown in Figure 7, AE at concentrations of 500, 1000, and 2000 mg/L all effectively promoted the gene expressions of BDNF, NCAM, and PCNA compared with control group (*P* < 0.01), and expressions of these genes had the most significant increase when concentration of AE was 1000 mg/L (*P* < 0.01).

## 4. Discussion

For the regeneration of injured peripheral nervous system (PNS), SCs play critical roles during this period via the synergetic effects with macrophages and neurons (repairing injured peripheral nerves: bridging the gap). Researches showed that in mammals, after peripheral nerve injury, SCs greatly reduce the expression of myelin [21–24]. At the first day after peripheral nervous damage, SCs digest intracellular myelin debris in the cavity to make the extracellular myelin debris exposure [25–27], which facilitate the macrophage phagocytosis and finally promote the axon regeneration [28]. Therefore, the proliferation of SC is crucial for the repair of peripheral nerve injury in our experiments. When the AE concentration was at 250–2000 mg/mL, AE significantly enhanced the proliferation of SCs. However, DP inhibited cell proliferation obviously when the drug concentration exceeded 4000 mg/L. Many researches had demonstrated the extracts of natural plants can accelerate cell proliferation. Normally, they play positive roles when they treat cells within an optimal concentration range. However, negative effects of the same extracts are often observed when the drug-treated concentration is too high [29, 30]. In order to further test the effects of AE on SCs cycles, SCs were treated with different concentrations of AE in vitro. AE at 500, 1000, and 2000 mg/L promoted the proliferation of SCs significantly, respectively, and elevated the cells counts in S phase and G2/M phase. In SCs, cells in G0/G1 phase were decreased; at the same time, cells in S phase and G2/M phase increased compared with control group. The mechanism of how AE promoted SC proliferation can be inferred. AE shortened the growth retardation, stimulate the cell to enter the active S phase from the stationary G0 phase, and increased the number in G2/M cells, which will lead to the proliferation of cells [31].

Neural factors, such as BDNF, NCAM, and PCNA, play very important roles for the peripheral nerve cell growth, development, and regeneration and maintain the survival of the nerve cells under normal condition. BDNF can promote regeneration of peripheral nerve, protect the damaged neurons and nerve cells, and maintain sensitivity to neurons [32]. BDNF also regulate and promote synthesis of adhesion in SCs [33, 34] and together BDNF and neurotrophic tyrosine receptor kinase type 2 (Ntrk2) are capable of activating the adhesion, angiogenesis, apoptosis, and proliferation pathways [35]. BDNF and NGF play essential roles in central nervous system [36]. NCAM is a kind of immunoglobulin secreted by SCs, and it also has a close relation to the axon regeneration, for example, combining L1 to form L1-NCAM complexes, which enhanced biological functions such as identification and adhesion [37, 38]. NCAM is a signal of glial cell-derived neurotrophic factor (GDNF) family receptors. GDNF activate NCAM and intracellular Fyn and FAK and promote the growth of nerve cells [28]. PCNA protein is essential for cell DNA synthesis, which is closely related to cell proliferation and can effectively reflect the activity of cell proliferation [39]. PCNA is a unique intranuclear protein, which plays a key role in DNA synthesis, damage healing, and regulation of cell cycle and usually is regarded as cell proliferation index [40]. Expressions of BDNF, NCAM, and PCNA in SCs treated by AE significantly increased compared to control group, and increased mRNA expressions of those were detected by RT-PCR. BDNF plays an essential role in promoting axonal regeneration and remyelination when SCs were transplanted into nerve injury lesions [41]. The overexpression of NCAM promotes neurite outgrowth and is implicated in myelination [42]. The results of PCNA protein upregulation were consistent with SC proliferation assay. These results showed that AE played an underlying role in peripheral nerve repair through promoting SC proliferation and stimulating SC to secrete neurotrophic factors. However, the active ingredients of* Angelica* such as polysaccharides, sodium ferulate, and volatile oil need to be further studied.

## 5. Conclusion

In this work, we demonstrated that AE promoted SC proliferation; furthermore, the results of cell cycle detection showed AE increased significantly DNA percentage in (G2 + S) phase. Simultaneously BDNF, NCAM, and PCNA protein expressions in SC significantly enhanced with the treatment of AE for 24 h or 48 h. Gene expressions of BDNF, NCAM, and PCNA also were upregulated markedly after drug treatment. Although the effects of AE on nerve repair have been already reported in many researches, we first studied the effects of AE on SC proliferation and cycle; moreover, many neurological factors which are related to nerve regeneration increased with AE treatment. Taken together, all of these results suggested potential application of AE in the clinical therapy of nerve injury.

## Figures and Tables

**Table 1:** Primers used for RT-PCR.

Gene	Serial	Primer (5′→3′)	Product size
BDNF	NM_001270638.1	Forward: CGGTATCAAAAGGCCAACTG Reverse: GTAGTTCGGCATTGCGAGTT	121 bp
NCAM	NM_031521.1	Forward: AACGGACTCCAAACCATGAC Reverse: TGGCTTTGCTTCTGACTCCT	122 bp
PCNA	NM_022381.3	Forward: TTGGAATCCCAGAACAGGAG Reverse: TTTGCACAGGAGATCACCAC	115 bp
*β*-Actin	NM_031144.3	Forward: TGTCACCAACTGGGACGATA Reverse: GGGGTGTTGAAGGTCTCAA	165 bp
